# Advances in the Welding of Aluminum Matrix Composites: A New Open Special Issue in *Materials*

**DOI:** 10.3390/ma15155335

**Published:** 2022-08-03

**Authors:** Cheng Gu, Guangjie Feng, Gaoyang Mi

**Affiliations:** 1 College of Materials Science and Engineering, Chongqing University, Chongqing 400045, China; fenggj@cqu.edu.cn; 2 School of Materials Science and Engineering, Huazhong University of Science and Technology, Wuhan 430074, China

“Advances in the Welding of Aluminum Matrix Composites” is a new open Special Issue of *Materials* that aims to publish original research and review papers on new scientific and applied research and to make great contributions to advances in the field of welding aluminum matrix composites as well as to the related synthesis, fundamentals, characterization, and application of these materials.

At present, energy conservation and emission reduction are the goals of unremitting efforts around the world and are required to prepare and process light- and high-strength materials [1]. As an advanced material that integrates both structure and function, aluminum matrix composites can be widely applied in the fields of aerospace, aviation, and automobile manufacturing, which have also become the main subjects of interest in the research and development of metal matrix composites [2].

However, the poor weldability of aluminum matrix composites is the most serious problem affecting their use. Thus far, research has been concentrated on the different welding characteristics of aluminum matrix composites, and the acquisition of high-strength welding joints will be the most important criterion for their wider application [3].

The research focus of the Special Issue “Advances in Welding of Aluminum Matrix Composites” includes but is not limited to the following: aluminum matrix composites; welding technology; simulation methods; microstructure; solidification processes; and molten pools.
